# The Emergence and Challenging Growth of the Bio-Ethanol Innovation System in Taiwan (1949–2015)

**DOI:** 10.3390/ijerph13020230

**Published:** 2016-02-19

**Authors:** Chao-Chen Chung, Siang-Cing Yang

**Affiliations:** Graduate Institute of Political Economy, National Cheng Kung University, Tainan 701, Taiwan; cindy330forever@gmail.com

**Keywords:** Taiwan, bio-ethanol, innovation system, policy

## Abstract

This study explores the bio-ethanol innovation system in Taiwan from the perspective of a technology innovation system (TIS). Taiwan is a newly industrialized country and is not currently a main producer of bio-ethanol. This study analyzes the evolution of bio-ethanol innovation system in Taiwan and places a particular emphasis on challenges that present policies face in the context of potential long-term bio-ethanol development. Through an evaluation of the consistency of the present research, technology, development and innovation (RTDI) policies as well as the influence of these policies on the functional dynamics of bio-ethanol innovation system, mechanisms prohibiting the system from flourishing are determined. It is suggested that the production of bio-ethanol in Taiwan would be achieved if the government: (1) fixes long-term targets for both domestic bio-ethanol development and emission reduction; and (2) comprehensively designs a set of interrelated RTDI policies in accordance with the functional pattern of the bio-ethanol innovation system and consistently implements these policies. If such measures were implemented, it is considered that the bio-ethanol innovation system in Taiwan would flourish.

## 1. Introduction

Bio-ethanol could become an industry of strategic significance, which not only possesses a tremendous potential to reduce the emission of green house gas (GHG) but at the same time can also contribute to the national economy and energy security. First-generation bio-ethanol is derived from a variety of feedstocks—typically agricultural crops such as sugarcane—whereas second-generation bio-ethanol is derived from energy crops such as miscanthus via the use of refining technologies. Bio-ethanol is now a broadly recognized liquid bio-fuel that could replace the use of fossil fuels on a global scale. However, productive regions of bio-ethanol are concentrated in areas driven by a variety of policy incentives. Brazil and the United States produced 86% of the world’s bio-ethanol in 2013, and since the 1970s, they have adopted policies to pursue energy security and industrial development; only in recent years have policies also considered the threat of climate change, as addressed by the United Nations Framework Convention on Climate Change (UNFCCC) in 1992 [1,2]. However, with the exception of China, other East Asian countries that have also launched bio-ethanol policies to mitigate global warming and to achieve energy security and economic growth are not the top producers of bio-ethanol, as shown in Table 1 [3].

Taiwan, as one of the newly industrialized East Asian countries that currently has limited development of bio-ethanol, has a great interest in further exploring the potential of this new form of energy. In 2013, Taiwan produced no bio-ethanol [4], but the existing Taiwanese innovation system of bio-ethanol re-emerged as early as 1949. The first generation of bio-ethanol was produced from molasses and aimed to achieve the production of cheap energy for the sugar industry. In the early 1980s, there was significant policy interest in new forms of energy in response to the oil crisis and the need for energy security. At this time, the Taiwanese government had no synthetic policies for the development of bio-ethanol, and bio-ethanol was simply used in the sugar industry. Then in 1997 when the Kyoto Protocol was opened for signature, the Taiwanese government launched further policies to accelerate the development of bio-ethanol, as an incentive to reduce global GHG emissions. These policies represented a strategic move to mitigate climate change and to contribute to the national economy and energy security. Nevertheless, the implementation of these policies in regulations and research schemes generated very limited benefits in relation to functions of the bio-ethanol innovation system.

The purpose of this article is to analyze the evolution of bio-ethanol innovation system in Taiwan and to particularly emphasize challenges that present policies face in the context of potential long-term development of bio-ethanol in this country. This undoubted potential would not be automatically achieved without new incentives. However, the establishment of new incentives is currently hindered for two reasons. First, as the long-term targets for maximizing industrial development and reducing emissions are not fixed, the uncertainty around the policies in place is high. Second, the set of inter-related policies promoted towards bio-ethanol cannot be consistently implemented. The analysis of the influence of government policies on the ups and downs of the bio-ethanol innovation system in Taiwan is the main aim of this article. 

This article is structured as follows: Section 2 reviews the theoretical background, Section 3 delivers the methodology, Section 4 shows the results of the evolution of policies and the innovation system of bio-ethanol, Section 5 presents a discussion and Section 6 concludes points made in the article.

## 2. Policies and Functional Dynamics of Bio-Ethanol Innovation System

A technology innovation system (TIS) is used to establish the foundation of our analytical framework. TIS can be described as “networks of agents interacting in a specific technology area under a particular institutional infrastructure to generate, diffuse and utilize technology” [5]. A TIS is comprised of three components: actors (firms and other organizations), networks and institutions. These three components are further described as follows. “Firms” can be embedded throughout the whole value chain, while “other organizations” refers to universities and other parts of the educational system, such as research establishments. Various types of “networks” exist between firms and other organizations. The learning networks transfer tacit and explicit knowledge while the advocacy coalition influences political agendas. Furthermore, “institutions” include both formal and informal ones: formal institutions are rules that are codified and implemented by authorities and deal with legal and regulatory aspects, and informal institutions represent rules related to social norms, culture and beliefs [6]. Institutions shape the interactions and networks between actors [7], and institutional change is at the heart of the process within which new technologies gain ground [8].

Government research, technology, development and innovation (RTDI) policies are among the most important institutions as they formulate interactions between actors and networks. To maximize the positive effects of policies on TIS, it is essential that the government aligns related incentive structures and employs consistent policy signals [9]. Consistency of RTDI policies according to Chung (2013) [10] can be further defined by two aspects. First, the policy objectives and instruments of a set of interrelated RTDI policies should not be contradictory to each other and should ideally be complementary, thereby offering no conflicting incentives to feed the dynamic development of the system. Second, the direction for the implementation of each single RTDI policy should be complementary to the objectives of the policy. Such consistent RTDI policies should appropriately match the functional dynamics of the TIS. The functions of a system are considered to be, “a contribution of a component or a set of components to a system’s performance” [11]. The functional pattern of the TIS, which is unique and is likely to differ from the ones of other TISs, would evolve over time [12]. In addition, the seven key functions of TIS have been synthetically identified by Bergek *et al.* (2008) [12], Negro and Hekkert (2008) [13] and Gosens and Lu (2013) [14].The definitions of the seven functions are shown in Table 2. Through analysis of system functions, governments can understand the inducement and blocking mechanisms related to technological development. In this way, RTDI policies can be adjusted to stimulate the weak and strengthen the proper functions of the TIS [15]. 

Several studies have analyzed the consistency of RTDI policies and their influence on the functional dynamics of bio-fuel TIS. In the field of gaseous bio-fuels, by expressing the empirical experiences of biomass gasification in the Netherlands, Negro *et al.* (2008) [15] showed how misalignment between institutional frameworks could negatively influence the functions of a bio-fuel innovation system. The Dutch government promoted a large research project in the northern Netherlands that was aimed to encourage innovations in bio-energy; after completion of preliminary studies of biomass gasification various actors had high expectations. However, despite initial enthusiasm the research project was determined to be inconsistently implemented and was thus eventually aborted. The policy of liberalization of the energy market, the objective of which was not complementary with the north Netherlands project, further reduced the interests of actors. As a result, the Dutch government was unable to realize a breakthrough in the development of gaseous bio-fuels. In contrast, the German government, as described by Negro and Hekkert (2008) [13], successfully supported the functional dynamics of biomass digestion via a satisfactory alignment of policies related to the needs of the biogas sector. In addition to setting up research programs and agencies, the government also focused on many system functions, such as the creation of legitimacy and market formulation and thus appropriately played the role of system builder. Moreover, in the field of cellulosic bio-ethanol, Gee and McMeekin (2011) [2] also expressed through the empirical experiences of the United States that synthetic RTDI policies are needed to effectively stimulate the functional dynamics of the innovation system. The Energy Policy Act of 2005 and the Energy Independence and Security Act of 2007 which called for 20% reduction of oil use within 10 years and required the increased use of “advanced bio-fuels”, such as cellulosic bio-ethanol, in fact secured a market for ethanol industry and clearly provided a strategic research direction for cellulosic bio-ethanol. Furthermore, the Biomass research and development (shortened to be R&D) instituted in the 2000 Biomass R&D Act promoted the development of cellulosic bio-ethanol capabilities and innovative activity in two major reverse salient: better feed stocks and more efficient conversion processes. As a result, the government of the United States properly stimulated the innovative and commercial activity in cellulosic bio-ethanol.

This study focuses on the Taiwanese bio-ethanol TIS as an empirical example. Experiences in Taiwan have so far been un-explored in the literature; only a few articles have set up initial discussions on the development of bio-ethanol in Taiwan. For example, Chen *et al.* (2013) [16] made one of the first attempts to investigate the Taiwanese technology trajectory of bio-fuels, including bio-ethanol. By analyzing patents registered in the European Patent Office, the authors of the study found that most Taiwanese patents related to bio-fuels were held by foreign direct investment (FDI) with a focus on chemical engineering and processing technologies. The domestic patent holders were predominantly public research institutes, such as Industrial Technology Research Institute (ITRI) and emphasized the second generation of bio-fuels. Only limited patents were held by private companies. Such technology trajectory indicated that the domestic bio-fuel technology was intensively guided by the government, which mainly encouraged the research of the second generation of bio-fuels, and domestic companies in fact lacked the incentive to invest in R&D in the related area. However, the article did not analyze the consistency of government policies and the influence of policies on the functional dynamics of the bio-ethanol innovation system in Taiwan. There have also been a limited number of studies done towards the simulation of bio-ethanol in Taiwan. For example, Su and Lee (2009) [17] assessed the lifecycle of sugarcane (as an agricultural crop) for its use in the production of bio-ethanol. From their perspective, a functional unit was defined as the bio-ethanol produced from 1 ha of sugarcane farmland in 1 year. As calculated by the two authors, the five stages in the lifecycle of bio-ethanol production included sugarcane cultivation, ethanol production, ethanol transportation, and energy use. Sugarcane grown in 1 ha absorbed 57, 103 kg CO_2_ per year. While the input to the entire lifecycle used diesel 177.5 L, chemicals 165.4 kg, electricity 2316 kWh, and water 257.9 m^3^, the output was CO_2_ 25,838 kg and biological oxygen demand (BOD) 1434.5 kg. Since most of the CO_2_ emitted when burning ethanol was simply the recycling of CO_2_ absorbed during plant growth, the net CO_2_ emissions were almost balanced. As sugarcane balanced CO_2_ emitted during the burning stage by its planting, it could thus be considered a suitable biomass feedstock. Nevertheless, the effects of indirect land use change (ILUC), which referred to be the transition from the carbon rich land like forest to cropland in order to meet the increasing demands for the production of bio-fuel feedstock, lay outside the scope of the lifecycle assessment, even though the ILUC effects could be potentially high [18]. Moreover, Su *et al.* (2015) [19] also investigated the water footprint of five energy and food crops: corn, sweet potatoes, sugarcane, sweet sorghum, and rice. The water estimated included the direct and indirect water usage during the cultivation stage of these crops, as well as the water consumption for bio-ethanol production. What was referred to as “water” contained green water (natural rain water), blue water (agriculture irrigation) and grey water (the use of water to dilute pollution). Based on the production of a ton of crop, the total water footprint of sweet potatoes was 96–118 m^3^, followed by sugarcane (187–204 m^3^ ), sweet sorghum (242 m^3^ ), corn (669–704 m^3^ ), and rice (1521–3288 m^3^ ). Besides, the consumption analysis based on the production of a liter of bio-ethanol showed that sweet potatoes were the most efficient, using 572–687 L water per liter of bio-ethanol, followed by corn (1808–1904 L water), sugarcane (2856–2909 L water), and sweet sorghum (3194–4394 L water). As green water composed more than 50% of the total water footprint of sweet potatoes, sweet sorghum, and sugarcane, these three crops which were more reliant on natural rainwater were more suitable for energy crops due to their low-input criterion. Meanwhile, sugarcane, which had the lowest grey water footprint among all crops, implied that sugarcane possessed the minimal impact of water pollution. As a result, government policies should prioritize sweet potatoes and sugarcane as the energy crops for bio-ethanol as they were better suited for the cultivation in the water-limited regions, such as Taiwan. However, among the various simulations and policy suggestions, these studies did not suggest any reasons why Taiwanese domestic companies are not strongly incentivized by the government to use agricultural crops or energy crops to produce bio-ethanol. Through the perspective of TIS, this study therefore aims to analyze the consistency of government RTDI policies and how the set of related policies will eventually shape the system functions of bio-ethanol in Taiwan; a system that once emerged but gradually faded out.

## 3. Methodology

We adopted the first-hand resources as the main resources of this study. The resources were collected through two rounds. Each round is described as the following.

The first round of desk research is completed with guidance from the TIS framework. We sketched the system structure and functioning based on “event sequence analysis“ as established by Negro *et al.* (2008) [15]. The events searched were those activities showing TIS functions (as shown in Table 3), and this was performed to gain an understanding of the influence of policies on the functional dynamics of the TIS. The scope of this search included various written sources, such as official statistics, government reports and news released by research organizations and universities. 

The second round of resource collection involved conducting expert interviews; this occurred after we had gathered information during the first round. We emphasized the consistency of RTDI policies and the influence of these RTDI policies on the functional dynamics of the bio-ethanol TIS. In this respect, expert interviews were conducted to further explore information that had not come to light while searching the written resources (such as actual implementation of policies and incentives of actors that legitimized the implemented policies). Interviewees were carefully selected and included those who had a deep involvement in bio-ethanol innovation and production. In total, 12 experts were interviewed: four government officials, six industrial experts and two academics. We used a semi-structured questionnaire, and several additional questions were adjusted and added in individual interviews to ensure that each of the interviewees provided the particular knowledge that we required.

## 4. Results

**Figure 1:**
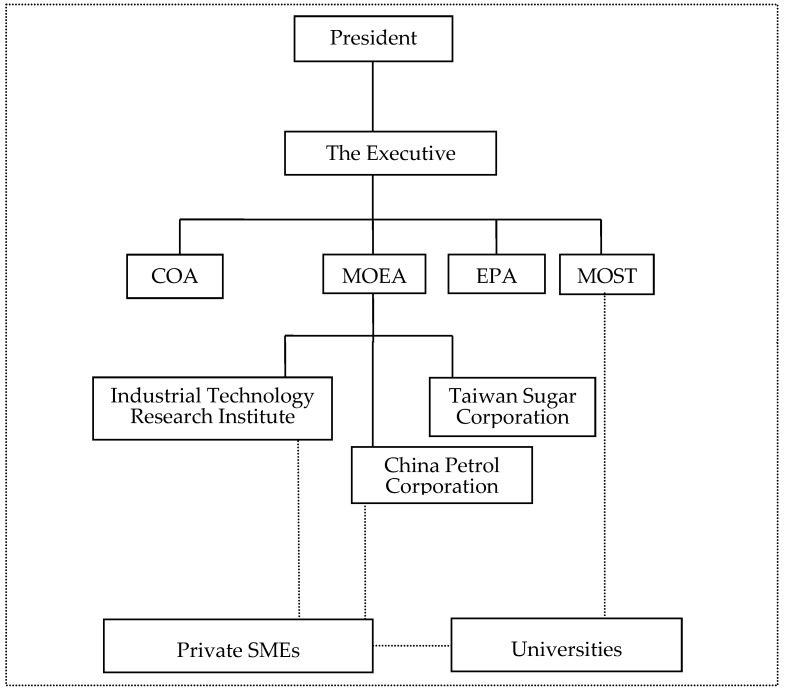
Institutions of the executive branch in Taiwan. Abbreviation: MOST = Ministry of Science and Technology (NSC = National Science Council), MOEA = the Ministry of Economic Affairs, COA = the Council of Agriculture, EPA=Environmental Protection Administration.

Early development of bio-ethanol in Taiwan dates back to the time of Japanese colonization in the 1930s. During this time, sugar cane was wildly cultivated in Taiwan; not only to satisfy the Japanese food demand but also because molasses, the residue from the sugar-refining process, was fermented to make bio-ethanol as a substitute for the petroleum required by the Japanese military. At the time, the Dai-Nihon Sugar Company (reorganized and renamed to be the Taiwan Sugar Corporation after 1949), which was established by the Japanese government, was the sole company controlling the sugar industry and the sole producer of bio-ethanol in the country. However, during World War 2, mass destruction of the sugar industry and plants occurred. Re-emergence of the bio-ethanol innovation system thus relied on efforts made by the government of the Republic of China (ROC, also known as the Taiwanese government). In the following sections, we divide the evolution of the bio-ethanol innovation system into three phases: re-emergence (1949–1997), suspension and re-evaluation (1998–2008) and industry stagnation (2009–2015). Since knowledge of the governmental structure is important for the understanding of the implementation of policies, Taiwanese government institutions are shown in Figure 1. Moreover, the influence of policies on the functions of the innovation system in each of the three phases is summarized in Table 4. In Table 4, following the operation of the system functions shown in Table 3, the positive value of policies is calculated as +1 and listed under “Positive Functions”, while the negative value will be calculated as –1 and listed under “Negative Functions“. If two policies have contradicting values and their effects reduced each other, we will calculate the value as 0 and listed under “Neutral Functions”.

### 4.1. Re-Emergence (1949–1997)

The bio-ethanol innovation system re-emerged in 1949 when Taiwan was acquired by ROC. Immediately following World War 2, the government mobilized resources to restore all sugar plants destroyed by war (F6), and bio-ethanol was simultaneously used by the sugar industry in their search for cheap energy (F4). The government’s fundamental purpose at this time was to stimulate exportation of cheap sucrose overseas to earn foreign exchange, which could then be invested into emerging manufacturing sectors. In support of policy guidance, the previous Dai-Nihon Sugar Company was reorganized to become the Taiwan Sugar Corporation (F7), which was the only sugar company and was public. Alongside the mass production of sucrose, the Corporation also intensively invested resources into the fermentation of molasses, which is the remains of the sugar-refining process, and produced molasses-based bio-ethanol as a first-generation bio-fuel (F6). However, 95% of manufactured bio-ethanol was consumed as edible alcohol, which supplemented the Corporation’s revenues, particularly when the price of sucrose was low. Only 5% of bio-ethanol was used as fuel for the Corporation’s locomotives, with the aim of reducing the cost of sugar production. In this respect, bio-ethanol was only considered to be a byproduct of the sugar industry, and the main business of the Corporation was the production and sales of sucrose.

The Arab Oil Embargo of 1973 (usually recognized as the First Oil Shock) prompted the Taiwanese government to establish more nuclear power plants as a substitute energy source for oil. In 1979, the OPEC Oil Crisis (the Second Oil Shock) led the government to consider using bio-ethanol as a potential oil replacement that could contribute to strengthening national energy security, and during the early 1980s, the Ministry of Economic Affairs temporarily allocated funding resources for the Taiwan Sugar Corporation to execute a research project on the “Application of biochemistry towards the fermentation of molasses” (F6) to avoid the potential fluctuation of oil prices for the third time. This project emphasized developing knowledge relating to improvements in the molasses fermentation process (F2); it is possible that such fermentation technology could be adopted for emergency use in a current potential new bio-ethanol plant. In addition, the Ministry of Economic Affairs funded the ITRI in 1989, with the aim of establishing a subordinate unit of the Institute of Energy and Minerals. This institute had the purpose of conducting research projects related to cellulosic ethanol (the second generation of bio-fuel), such as fermentation of residues from the food processing industry (F2 and F6). Universities, such as National Taiwan University, were then funded by the Ministry of Economic Affairs to perform experiments and develop knowledge of fertilizers for cellulosic ethanol (F2 and F6). However, at this time the Ministry of Economic Affairs only recognized bio-ethanol as a potential replacement for oil that could be used when importing oil was problematic. Therefore, when a third oil crises failed to occur the government lost interest in funding research projects for bio-ethanol (−F6), and the drive for knowledge accumulation in relation to bio-ethanol also faded, particularly that related to second-generation bio-ethanol (F2).

In the 1990s, the Taiwan Sugar Corporation continued to invest in the production of bio-ethanol using molasses as a by-product of sugar industry (F6). In addition, by the mid-1980s, the Taiwanese economy has been thoroughly transformed into an export-oriented manufacturing economy and no longer relied on the exportation of sugar for foreign exchanges. Taiwanese sugar, which used to compete in the international market on a price-base, was no longer cheap enough to compete with cheap sucrose from Brazil and Cuba and therefore several sugar plants were shut down during the mid-1980s and the 1990s. The production of domestic molasses was extensively decreased to the extent that, in the 1990s molasses needed to be imported for part of the production of bio-ethanol. Then, with the declining production of bio-ethanol, the Taiwan Sugar Corporation also needed to purchase diesel for locomotives and gradually suspended investment of resources into the production of bio-ethanol (−F6).

### 4.2. Suspension and Re-Evaluation (1998–2008)

The Kyoto Protocol, which was opened for signature in 1997, introduced the principle of reducing GHG and became the new incentive for the government to support the development of bio-ethanol production. Bio-ethanol was gradually supported by policies as the industry, which could contribute to a better future of the national economy and improved energy security, as well as help the nation to fit its responsibility of reducing emissions (F4). In response to the opening signature of the Kyoto Protocol, the Taiwanese government held the first National Energy Conference in 1998. This conference concluded by deciding on a target that emissions of CO_2_ would be reduced to the level of the year 2000 by the year 2020. The Conference also decided that new forms of energy should be made available and that by the year 2020 these new energies should account for 1%–3% of the total energy used. Following this conference, the Ministry of Economic Affairs funded and then published a research report named the “Research and Development of New and Clean Energy Report” (1999), and the National Science Council also funded and released a research report entitled the “Long-term Development of Energy Technology Plan” (F2 and F6). These two reports emphasized the potential for mass production of second-generation cellulosic bio-ethanol, and the message contained within meant that the production of cellulosic bio-ethanol as a new form of energy was systematically evaluated for the first time. Subsequently, when the Kyoto Protocol came into effect in 2005, the government convened the second National Energy Conference in the same year, the conclusion of which was that it would be difficult to achieve a reduction in CO_2_ emissions to levels of the year 2000 by the year 2020. Therefore, the government established a new schedule for reducing CO_2_ emissions in two stages. It was deemed that during the first stage, from 2005 to 2025, the average growth rate of CO_2_ emissions should be 1.5% (slightly higher than 1%, the standard of Organization for Economic Co-operation and Development (OECD), countries); during the second stage, after 2025, the Taiwanese growth rate of CO_2_ should then be reduced to 1% (the same level as OECD countries). This second National Energy Conference also reconfirmed that the government would encourage the production of cellulosic ethanol from non-edible energy crops. Therefore, although the conclusion of the second Conference decreased the target established by the first Conference, the vision of reducing GHG and achieving sustainable development was highly emphasized and expected (F4).

In accordance with the direction concluded by the two National Energy Conferences, a concrete regulation policy was determined for bio-ethanol. This regulatory policy played the role of formulating a market for bio-ethanol (F5). The Petroleum Administration Act (known as “the Act”) was firstly legislated in 2001 and was then further amended in 2008 to add new clauses stating that fossil fuels should blend with bio-fuels by a fixed ratio (as shown in Table 5). In 2007, on the basis of this Act the Ministry of Economic Affairs launched the project known as the “Bio-ethanol Execution Plan” (known as “the Execution Plan”), which formulated an initial market for bio-ethanol (F5). The original Execution Plan divided the adoption of bio-ethanol into three stages. The first stage ran from early 2007 to the end of 2008, and this implemented the first sub-project of the Execution Plan, known as the “Green Public Vehicle Pilot Plan”. In accordance with this plan, it was deemed that public vehicles in Taipei City must refuel E3 petrol which referred to the ratio of normal petrol to bio-ethanol was 97%:3%, and eight designated petrol stations were to provide E3 petrol for all private vehicles volunteering to refuel. The provision of domestic bio-ethanol should be prioritized, and only the volume lacking was to be supplemented by imports. However, the Taiwan Sugar Corporation had already suspended the production of molasses-based bio-ethanol in 2003, and therefore, in 2007,the Council of Agriculture in cooperation with the Ministry of Economic Affairs subsidized a public company, the Taiwan Tobacco and Liquor Corporation, to supply initial domestic demands by producing a small amount of first-generation bio-ethanol by fermentation of sweet potatoes. It was planned that E3 petrol would be gradually adopted nation-wide in the second and third stages, from 2009 to 2011, and this is further discussed in Section 4.3.

At this time, the Taiwan Sugar Corporation continued to be regarded by the government as the most important company involved in the mass production of bio-ethanol in the long-term, despite the fact that the Corporation had already terminated production of bio-ethanol by molasses in 2003 because of the high costs involved and the low revenues from the product. Nevertheless, to support the implementation of the Execution Plan (F7), the Corporation invested in a new research project in 2007 (F6), with the aim of evaluating the possibility of re-producing bio-ethanol using sugar cane juice and bagasse(F2), as well as determining the feasibility of establishing a new bio-ethanol plant (F1). 

### 4.3. Industry Stagnation (2009–2015) 

The progress of the Kyoto Protocol gave the government continued incentive to support the production of bio-ethanol, with the aim of accelerating economic development, providing energy security and reducing GHG. In fact, in response to the Copenhagen Accord in 2009, the third National Energy Conference was summoned in the same year, and the government once again adjusted its emissions target stating that from 2016 to 2020 emissions of CO_2_ should be reduced to those of the 2008 level and that the 2000 level need only be achieved by 2025. It was also decided that the production of second-generation cellulosic ethanol from domestic agricultural residues or non-edible energy crops should be encouraged in relation to bio-ethanol production. The Conference also proclaimed that the government would launch the “National Energy Program” (hereafter referred to as “the National Program”) to support the innovation of renewable energies, including bio-ethanol. Moreover, at the beginning of 2015, the government held the fourth National Energy Conference not only to respond to the Copenhagen Accord but also to solve the problem that the development of new nuclear plants was totally suspended in mid-2014. In the Conference conclusions, the target for emissions reductions was then further altered, and it was determined that CO_2_ emissions should be reduced to those of the 2005 level by 2020 and that National Appropriate Mitigation Actions (NAMAs) should be taken to reduce emissions of GHG by this date to at least 30% of Business As Usual (BAU). Furthermore, the government declared that it would continuously support the development of second-generation bio-ethanol prioritizing the utility of domestic agricultural residues. In short, the vision of sustainable development was re-confirmed, and it was declared that second-generation bio-ethanol would play an important role in fulfilling this vision (F4). 

The directions dictated by the National Energy Conferences promoted concrete policies of regulation and the National Program. While regulations on the basis of the Act aimed to formulate a market for bio-ethanol (F5), the National Program was launched to achieve policy objectives of energy “security, efficiency and cleanliness”, as shown in Table 5. Among the various funding subjects of the National Program, it was determined that bio-ethanol was to be supported as an “alternative energy industry”, which would “not only help slow global warming and reduce GHG emissions”, but would also “bring about another wave of economic development based on green energy industries in Taiwan”. The National Program was jointly funded by the budgets of the Ministry of Economic Affairs and the Ministry of Science and Technology (F6), and it encouraged the development and diffusion of knowledge between universities and firms (F2 and F3).

However, the regulation policy (the Execution Plan) was not implemented as intended in the original plan. According to the original design of the Execution Plan, the Ministry of Economic Affairs were to execute its second sub-project known as “Metropolitan Area E3 Plan” from early 2009 to the end of 2010 and provide E3 petrol for all vehicles in Taipei City and Kaohsiung City. Furthermore, in the third stage of the plan, from early 2011 onwards, E3 petrol was to be supplied for all vehicles nationwide. In addition, the original Execution Plan declared that domestic bio-ethanol would be adopted first and that imports would only be acquired as a supplement. However, after 2008, implementation of the original Execution Plan faltered; by 2007 it had been discovered that the cost involved in producing the small amount of domestically produced bio-ethanol from fermented sweet potatoes was higher than the cost of imported product (−F2). As one expert explained, the Ministry of Economic Affairs therefore expressed concerns that motorcycle users, who mostly belonged to a lower socio-economic group, may not be able to afford E3 petrol as it was more expensive than pure petroleum. As a result, between the years of 2009 and 2015, E3 petrol was only used in public vehicles within Taipei City and Kaohsiung City, and all the bio-ethanol used was imported. It is thus considered that this distorted implementation of the Execution Plan caused the complete crash of the domestic bio-ethanol market (−F5).

The implementation of the Execution Plan was not legitimized by Taiwan Sugar Corporation (−F7). The Corporation, which had once submitted a proposal to construct a new bio-ethanol plant to the Executive Yuan (and which had achieved approval in 2011), decided to withdraw its proposal in mid-2014 (−F1). Indeed the Corporation didn’t actually involve in any production of bio-ethanol. 

The National Program was still promoted even when the implementation of the Execution Plan was distorted. The National Program subsidized research into cellulosic ethanol in universities and firms and promoted knowledge flow between the two actors (F2, F3 and F6). In 2012, under the funding scheme of the National Program, the network created between firms (China Petroleum Corporation and the Taiwan Sugar Corporation) and universities (National Chung Hsing University and National Taiwan University) jointly carried out a research project known as the “Bio-ethanol Demonstration Program in Tainan City”, which produced the first volume of cellulosic bio-ethanol from fermented domestic bagasse and straws using domestic enzyme hydrolysis (F2 and F3). Although the bio-ethanol produced by the research project was more expensive than imported bio-ethanol and normal petrol, temporary subsidies from the National Program were provided to maintain it at a cheaper price than the normal petrol (reduced NT$ 3.5/US 0.1 per liter). Unfortunately, following delivery of the first volume of bio-ethanol to a petrol station owned by the Taiwan Sugar Corporation, subsidies were terminated when the volume was sold out, and the government showed no interest in subsidizing bio-ethanol in the long-term (−F6). 

However, the launch of the National Program still incentivized several newly established small and medium enterprises (SMEs) to invest in research on cellulosic ethanol (F1, F2 and F6). Without accumulation of sufficient knowledge, these SMEs transferred technologies from universities, public research institutes and foreign companies (F3) and invested in the process innovation of mass production of cellulosic ethanol, particularly anhydrous butanol (F2 and F6). For example, Cosmo Ltd, which was established in 2011, transferred enzyme hydrolysis technology from Mother Cosmo (Japan) and introduced innovations for mass production in Taiwan. This company also cooperates with National Chung Hsing University, through funding for the National Program, to carry out research on collection systems for agricultural residues in Taiwan. In addition, Ding-Tang, the spin-off of ITRI set up in 2014, heavily invested in the mass production of bio-butanol from agricultural residues as a replacement for fossil butanol. Nonetheless, implementation of the Execution Plan, which only applied to E3 bio-ethanol in public vehicles in Taipei City and Kaohsiung City and which only used imported bio-ethanol, actually discouraged domestic SMEs from investing in domestic production (−F5 and −F6). As explained by one industrial expert, SMEs made no further investment in the production facilities because they could not expect a positive response from the domestic market for the bio-ethanol produced (−F4). In addition, another expert explained that since there was no recycling system of agricultural residues, it would be too expensive for companies to obtain domestic raw materials for cellulosic ethanol (−F4). As a result, even though domestic SMEs actually had the necessary technology to develop production, they instead focused their interest in licensing companies rather than investing in domestic production, and technologies were then transferred to foreign countries.

## 5. Discussion

The evolution of the bio-ethanol innovation system in Taiwan has been deeply shaped by the government RTDI policies. According to the summary displayed in Table 4, before 1997, the system functions were only weakly fulfilled; government resources were mobilized to produce first-generation bio-ethanol (F6) mainly in relation to providing cheap energy for the sugar industry (F4). In response to the oil crisis in the early 1980s and to sustain energy security, limited occasional research funding was then given to the Taiwan Sugar Corporation and universities (F6) to conduct research on the development of cellulosic ethanol (F2). After 1997, with the progress of Kyoto Protocol, the need for bio-ethanol production was significantly driven by the vision of sustainable development, in which bio-ethanol played the role of an industry that could contribute to the national economy and energy security and could reduce GHG emissions. In comparison with the previous period, after 1997 a number of synthetic policies were launched to stimulate the growth of bio-ethanol. However, the development of the bio-ethanol innovation system remained stagnant until the end of 2015. Throughout this period, conclusions from the four National Energy Conferences, which set up targets for CO_2_ reduction, were not fixed and instead were continuously revised. In addition, the National Energy Conference only announced overall targets for all sectors, but did not deliver detailed targets for each particular sector. The function of the Guide on the Search was thus weakened (−F4).

Furthermore, the bio-ethanol policies didn’t remain consistent through 1997 to 2015 and had limited appropriate support to the functional dynamics of the TIS. In the period 2000–2008, the regulation policy (the Execution Plan) gradually formulated a market for E3 petrol (F5). This regulation gained the support of the Taiwan Sugar Corporation (F7), which was willing to invest in a new research project (F2 and F6) and evaluated the possibility of establishing new bio-ethanol plant (F1). However, the Execution Plan and the National Program were not consistently implemented in the period 2009–2015. Following the Act, the policy objectives of the Execution Plan to “develop renewable domestic energy” that “comprehensively combined the development of agriculture, environment and industry” were actually complementary to the policy objectives of the National Program, whose policy objectives were to ensure “security, efficiency and cleanness” of energy and subsidized bio-fuels as an “alternative energy industry”. In other words, both policies supported bio-ethanol out of economy, security and the reduction of emissions. However, implementation of the Execution Plan was distorted. It had originally prioritized production of domestic bio-ethanol and intended the Formulated Market to be nationwide (F5), but ultimately E3 petrol was only used on a small scale and only imported goods were used (−F5). Without the promise of a future market, companies such as the Taiwan Sugar Corporation and new SMEs were unwilling to continue along the technological trajectory to which they had been committed (−F1 and −F6). Even though the National Program continued Mobilizing Resources (F6) and positively encouraged universities and firms in relation to Developing Knowledge (F2), Diffusing Knowledge (F3) and even Entrepreneurship Activities (F1), the positive effects of the National Program were reduced by the distorted implementation of the Execution Plan. As a result as supported in interviews with industrial experts, firms funded by the National Program have only been willing to transfer their knowledge overseas. Interviews with academics show their positive expectations in relation to developing knowledge of bio-ethanol production. However, the conservative attitudes shown by the government interviewees indicate that the government is only willing to support knowledge development and diffusion, without removing barriers to market formulation. Therefore, as no companies are willing to invest in domestic production, it is evident that the two policies together have not been successful in supporting the bio-ethanol industry, an industry that would significantly contribute to national economy, energy security and help the nation to reduce emissions.

In summary, the Taiwanese government promoted bio-ethanol policies to respond to Kyoto Protocol and to accelerate domestic industrial development and energy security, yet with limited achievements. It is therefore considered that there should be at least two policy recommendations implemented in the bio-ethanol TIS to solve the current blocking mechanisms pertaining to policies. Firstly, the government should fix the long-term targets for both the domestic bio-ethanol development and for reducing emissions. Nowadays, there is no fixed target for either the industry or the emissions. In fact, not only the fixed overall national target of emission is needed but also detailed targets for each sector, such as transportation. The detailed target of transportation should be set up in compliance with domestic production and innovation and supported by long-term policies. The constant policy commitment will increase the certainty of companies’ investments. As described by Gallagher *et al.* (2012) [9], companies only respond to credible policies, and volatility can accelerate knowledge depreciation. Secondly, the government should design a comprehensive set of related RTDI policies according to dynamics of the functional pattern of bio-ethanol TIS and to consistently implement these policies. Current regulations and R&D policies are not implemented in a complementary direction. If the Taiwanese government still recognizes the potential of producing second-generation bio-ethanol which would not follow food to fuel pathway, it needs not only to re-align implementation of the existing policies, but needs to initiate new policies that complement the existing ones to effectively support the functional dynamics of the bio-ethanol innovation system. As we have mentioned in Section 2, to support cellulosic bio-ethanol, the government of the United States promoted the regulation policies, such as the Energy Policy Act and the Energy Independence and Security Act, to formulate a strategic research direction (F4) and a market for cellulosic bio-ethanol (F5). Besides, the government of the United States also launched R&D policies like Biomass R&D Act to mobilize resources to accelerate knowledge development and diffusion (F6, F2, and F3). When the government stimulus for innovative and commercial activity increased, entrepreneurship activities arose (F1); for example, DuPont established a Cellulosic Ethanol Facility in Nevada, which is expected to lead the way for the commercialization of cellulosic bio-ethanol [20]. In the case of Taiwan, we suggest a recycling system for agricultural residues should be established to reduce the cost of cellulosic bio-ethanol (F6), and such new recycling policies should be supplemented by the Act. This would steadily expand the market (F5) and long-term R&D funding for mobilizing resources (F6) in the development and diffusion of knowledge (F2, and F3). With positive expectations for market expansion (F4), it is considered that legitimate interest from stakeholders, particularly companies, would increase (F7), as would entrepreneurial activities (F1).

## 6. Conclusions

This article explores the bio-ethanol innovation system in Taiwan from the perspective of TIS. Taiwan is a newly developed industrialized East Asian country, but it is not currently a main producer of bio-ethanol. By evaluating the consistency of present RTDI policies, as well as the influence of these policies on the functional dynamics of bio-fuel TIS, mechanisms blocking the TIS are determined. It is suggested that the government fix targets for both industrial development and emission reductions and that it should steadily support the production of bio-ethanol in the long-term. It is also suggested that a set of related policies should be consistently implemented with legitimate interest from stakeholders. If the government considered these suggestions, it is believed that the bio-ethanol innovation system in Taiwan would grow.

## Figures and Tables

**Table 1:** Bio-fuels global production (top 16 countries and EU-27), 2013.

Country	Fuel Ethanol	Biodiesel	HVO	Total	Comparison with Total Volumes Produced in 2012
Billion liters					
United States	50.3	4.8	0.3	55.4	+1.2
Brazil	25.5	2.9	-	28.4	+4.1
Germany	0.8	3.1	-	3.9	+0.2
France	1.0	2.0	-	3.0	+0.1
Argentina	0.5	2.3	-	2.7	−0.3
The Netherlands	0.3	0.4	1.7	2.5	No change
China	2.0	0.2	-	2.2	−0.1
Indonesia	0.0	2.0	-	2.0	+0.2
Thailand	1.0	1.1	-	2.0	+0.5
Canada	1.8	0.2	-	2.0	+0.1
Singapore	0	0.93	0.9	1.8	+0.9
Poland	0.2	0.9	-	1.2	+0.3
Colombia	0.4	0.6	-	0.9	No change
Belgium	0.4	0.4	-	0.8	No change
Spain	0.4	0.3	-	0.7	−0.2
Australia	0.3	0.4	-	0.6	No change
EU-27	4.5	10.5	1.8	16.8	1.3
World	87.2	26.3	3.0	116.6	7.7

HVO is the abbreviation of “hydrotreated vegetable oil” which is also known as “renewable diesel” produced from used cooking oils, fats, and vegetable oils [3]. Source: [3].

**Table 2:** Definitions of TIS functions.

System Functions	Definition
Function 1: Entrepreneurship Activities	Entrepreneurs perform market-oriented experiments and make efforts to develop marketable technological applications. Entrepreneurship Activities are indicated by new entrants and different types of technological applications. Entrepreneurs can be private or public enterprises.
Function 2: Knowledge Development	Accumulation of knowledge is the precondition for the birth of innovation. Research and the development of knowledge can be undertaken by different actors within the innovation system, including academics in universities, research institutes and companies.
Function 3: Knowledge Diffusion	The network between different actors facilitates the exchange of information. Through knowledge exchange activities, such as communication and technology transfer, knowledge spills over in the TIS.
Function 4: Guidance of Search	This function covers mechanisms that have an influence on the direction of search within the TIS, including different competing technologies, applications, markets and business models. Guidance can be taken from the institutional form of policy targets, but is also realized by the expectations of different actors.
Function 5: Market Formulation	Governments can create niche markets for new technologies, especially when the new technologies cannot exceed incumbent technologies. The creation of markets is necessary to stimulate innovation.
Function 6: Resource Mobilization	Technology innovation requires the inputs of capital and human resources. Resource mobility could be fulfilled through entrepreneurial investments or government supported funding.
Function 7: Legitimation	Legitimation refers to social acceptance and compliance with relevant institutions. The responses of actors to policies, agreement, or opposition are revealed through lobbying activities or compliance with policy implementation. Such responding activities could be practiced by particular interest groups or individual actors.

Sources: [12,13,14,15].

**Table 3:** Operation of system functions.

System Functions	Even Category	Sign/Value
Function 1: Entrepreneurship Activities	Project started	+1
	Project stopped	−1
Function 2: Knowledge Development	Desktop/Assessment/Feasibility studies, reports	+1
Function 3: Knowledge Diffusion	Conferences and technology transfer	+1
Function 4: Guidance of Search	Clear policy directions, positive expectations of bio-fuels	+1
	Confused policy directions, negative expectations on bio-fuels	−1
Function 5: Market Formulation	Regulation expanding the market of bio-fuels	+1
	Regulation that once expanded the market of bio-fuels diminished	−1
Function 6: Resource Mobilization	Government subsidies and R&D fundingCompany’s investments	+1
	Government expressed lack of subsidies and R&D funding Company withdrew or suspended investments	−1
Function 7: Legitimation	Successful lobby by actors to improve technical, institutional and financial conditions for bio-fuel Positive willingness of actors to comply with the policy implementation	+1
	Lobby of actors for improvement received no positive responses from related stakeholders or the government Successful lobbying activities from a rival coalition Unwillingness of actors to comply with policy implementation	−1

Source: [13].

**Table 4:** The influence of policies in system functions.

Phase	Positive Functions	Neutral Functions	Negative Functions
Phase1: re-emergence (1949–1997)	F2, F4, F6	F1, F3, F5, F7	None
Phase 2: suspension and re-evaluation (1998–2008)	F1, F2, F5, F6, F7	F3	F4
Phase 3: industry stagnation (2009–2015)	F2, F3	F1, F6	F4, F5, F7

**Table 5:** Detailed content of regulations and R&D policies.

Policy Names	Policy Type	Policy Contents
The Petroleum Administration Act	Regulation	Ministrie: MOEA. Year of promotion: 2001–2014. Policy objectives (Article 1): “The Petroleum Administration Act (henceforth the Act) is being instituted to promote the sound development of the oil industry, to safeguard the production and sales of oil, to ensure the steady supply of oil, to enhance people's livelihoods and to develop the national economy while at the same time giving equal consideration to environmental protection”. Policy instruments (Article 38-1): “The central competent authority can determine the fixed blending ratio of alcohol or ester to gasoline and diesel in accordance with the actual implementation schedule and within the scope and method determined by the oil refinery and oil importer. The aforesaid blending ratio of alcohol or ester to gasoline and diesel, the actual implementation schedule, scope and method should be promulgated by a central competent authority”.
The Execution Plan of Bio-ethanol	Regulation	Ministries: MOEA. Year of promotion: 2007–2014. Policy objectives: to “develop domestic renewable energy comprehensively in combination with the development of agriculture, environment and industry”. Policy Instrument: blending ratio between gasoline and bio-ethanol.
Technology Development Program	R&D	Ministries: MOEA. Year of promotion: 1998–2008 (towards bio-fuels). Policy objectives: “to initiate R&D innovation, break ground in relation to industrial technology development and reinforce national competitiveness”. Policy instruments: funding.
The National Science and Technology Program—Energy	R&D	Ministries: MOST and MOEA. Year of promotion: 2009–2014. Policy objectives: “security, efficiency and clean energy”. Expected Benefits. **Improve energy efficiency and reduce dependence on imported energy.** **Improving the international competitiveness of alternative energy industries:** The development and promotion of clean energy is used to reduce the use of fossil fuels, not only to help slow global warming and reduce GHG emissions, but also to help realize the policy goal of returning Taiwan’s 2025 CO_2_emissions to 2000 levels, while simultaneously cultivating the international competitiveness of domestic alternative energy industries to bring about another wave of economic development based around green energy industries in Taiwan. The development of alternative energy technologies will also be used to establish industries for a power-to-grid storage system incorporating electric vehicles, **non-food feedstock production technology**, **innovative catalytic trans esterification technology for bio-diesel production** and high C/P PV cell/module technology industries. **Develop smart grid technology industry and help build a smart grid system in Taiwan.** **Develop smart offshore wind power and ocean energy technology industries, strengthen the development of offshore wind and ocean power and realize a domestic power production of 2.1 Billion kWH and carbon reductions of 1.3 Million tonnes by 2020.** **Development of clean geothermal energy.** Policy instrument: funding

Abbreviation: MOST= the Ministry of Science and Technology, MOEA= the Ministry of Economic Affairs.
